# Predictive value of NRI, NLR, PLR and LMR for therapeutic efficacy and prognosis in advanced rectal cancer patients treated with PD-1/PD-L1 immunotherapy

**DOI:** 10.3389/fsurg.2026.1809691

**Published:** 2026-05-28

**Authors:** Mengli Gao, Zhe Gao, Yanan Wang, Wenguang Song

**Affiliations:** Department of Oncology, Tangshan Gongren Hospital, Tangshan, Hebei, China

**Keywords:** immunotherapy, Lymphocyte-to-monocyte ratio, Neutrophil-to-lymphocyte ratio, Nutritional risk index, platelet-to-lymphocyte ratio, Predictive Value, rectal cancer

## Abstract

**Objective:**

The purpose of the study was to investigate the predictive value of the Nutritional Risk Index (NRI), Neutrophil-to-Lymphocyte Ratio (NLR), Platelet-to-Lymphocyte Ratio (PLR), and Lymphocyte-to-Monocyte Ratio (LMR) for the prognosis and effectiveness of immunotherapy in patients with advanced rectal cancer.

**Methods:**

Retrospective analysis of 172 advanced rectal cancer patients undergoing immunotherapy (Feb 2020 - Feb 2023) was performed. The optimal cut-off values of each indicator were determined by receiver operating characteristic (ROC) curves. The *χ*^2^ test was used to analyze the correlations between indicators and clinicopathological characteristics, objective response rate (ORR), and disease control rate (DCR). Kaplan–Meier method was used to draw survival curves, and Log-rank test was used to compare the differences in progression-free survival (PFS) and overall survival (OS). Cox proportional hazards regression model was used to screen independent influencing factors for efficacy and prognosis.

**Results:**

The optimal cut-off values of NLR, PLR, LMR, NRI were 2.50, 142.23, 3.92, 50.45. Efficacy analysis revealed that the ORR and DCR in the low NLR, low PLR, high LMR, and high NRI groups were significantly higher than those in the corresponding groups (all *P* < 0.05). Survival analysis indicated that patients with high NLR and high PLR had significantly shorter median PFS and OS (all *P* < 0.001). Multivariate Cox regression analysis confirmed that TNM stage Ⅳ (HR = 2.014) and high NLR (HR = 2.689) were independent risk factors for shortened PFS; TNM stage Ⅳ (HR = 1.923), poorly-undifferentiated tumor (HR = 1.786), and high NLR (HR = 2.519) were independent risk factors for shortened OS.

**Conclusion:**

These indicators correlate with immunotherapy efficacy/prognosis. Among them, NLR≥2.50 is an independent biomarker for poor efficacy and prognosis, providing references for clinical treatment individualization.

## Introduction

Rectal cancer is a common gastrointestinal malignant tumor with high incidence and mortality worldwide (1). For patients with advanced or metastatic rectal cancer, treatment options are limited, and the prognosis remains poor. In recent years, immunotherapy represented by PD-1/PD-L1 inhibitors has brought breakthroughs in the treatment of advanced rectal cancer, and significantly improved the survival prognosis of specific patient populations (2). However, the clinical benefit of immunotherapy in rectal cancer presents a high degree of heterogeneity. Tumors with mismatch repair-deficient/microsatellite instability-high (dMMR/MSI-H) account for approximately 5% to 10% of advanced rectal cancer cases; in this population, the objective response rate (ORR) of PD-1/PD-L1 inhibitor monotherapy can reach 30% to 50%, and multiple clinical studies have confirmed its long-term survival benefit (3, 4). In contrast, for patients with mismatch repair-proficient/microsatellite stable (pMMR/MSS) tumors, which make up the vast majority of advanced rectal cancer cases, the ORR of single-agent immunotherapy is less than 10%, and combination treatment strategies are usually required to enhance therapeutic response (5). Therefore, the identification of simple and reliable biomarkers to screen populations who can benefit from immunotherapy and predict treatment efficacy and prognosis is of critical clinical importance for optimizing treatment strategies and improving the survival benefit of patients.

Tumor patient prognosis, treatment tolerance, and immune function are all strongly correlated with nutritional health. The Nutritional Risk Index (NRI), a nutritional evaluation tool based on changes in weight and serum albumin, has been shown to accurately predict the effectiveness of treatment and survival outcomes for patients with a variety of malignant tumors (6, 7). Moreover, tumor immune evasion, tumor development, and treatment resistance are all significantly influenced by inflammatory responses connected to tumors. Neutrophil-to-Lymphocyte Ratio (NLR), Platelet-to-Lymphocyte Ratio (PLR), and Lymphocyte-to-Monocyte Ratio (LMR) have demonstrated potential value in predicting the effectiveness and prognosis of immunotherapy in various tumors, including lung cancer and gastric cancer, as straightforward and affordable indicators reflecting systemic inflammatory responses (8, 9). Nevertheless, studies on the predictive value of NRI combined with inflammatory indicators for the efficacy and prognosis of immunotherapy in rectal cancer patients are still incomplete, and their synergistic predictive efficacy needs further verification. Based on this, this study intends to investigate the correlations between pretreatment NRI, NLR, PLR, LMR and the efficacy, progression-free survival (PFS) and overall survival (OS) of immunotherapy in rectal cancer patients, and screen independent predictive biomarkers for immunotherapy benefit, so as to provide scientific basis for clinical screening of populations benefiting from immunotherapy and formulating individualized treatment plans.

## Materials and methods

### Study population

172 patients with advanced rectal cancer who underwent PD-1/PD-L1 inhibitor immunotherapy at Tangshan Gongren Hospital from February 2020 to February 2023 were enrolled retrospectively. Inclusion criteria: (1) Age > 18 years. (2) All enrolled patients were histopathologically confirmed with rectal adenocarcinoma; (3) Tumors were staged as stage Ⅲ to Ⅳ according to the 8th edition of the American Joint Committee on Cancer (AJCC) Rectal Cancer Staging System (10), and patients were comprehensively assessed by a multidisciplinary team (MDT) consisting of gastrointestinal surgery, medical oncology, radiology, radiation oncology, and pathology departments, and confirmed to be ineligible for radical surgical resection; (4) Received at least 2 cycles of PD-1/PD-L1 inhibitor monotherapy or combined therapy (combined with chemotherapy, targeted therapy, etc.); (5) Completed peripheral blood routine and serum albumin detection within 1 week before treatment, with complete clinicopathological data and follow-up data; (6) Presence of measurable tumor lesions (according to RECIST 1.1 criteria) (11). The judgment criteria for ineligibility for radical surgical resection were defined as follows:① Locally advanced tumor invading adjacent organs/pelvic wall, or accompanied by extensive regional lymph node metastasis, which failed to achieve R0 resection of the primary tumor;② Accompanied by distant metastasis that was not suitable for R0 resection, including extensive multi-organ metastasis, diffuse peritoneal carcinomatosis, etc.;③ Patients with severe underlying comorbidities, who were assessed by the MDT to be intolerant to the trauma of radical surgery and perioperative risks. Exclusion criteria: (1) Complicated with other malignant tumors; (2) Presence of active infection, autoimmune diseases, or use of glucocorticoids and other immunosuppressants before treatment; (3) Severe liver and kidney dysfunction, coagulation disorders, or other serious underlying diseases; (4) Incomplete clinical data or lost to follow-up.

Sample size estimation: According to the results of preliminary pre-experiments, assuming the AUC of NLR=0.65, *α*=0.05, *β*=0.10, the minimum sample size required was calculated using the ROC curve sample size formula *n* = (Z*_α_*_/2_ + Z*_β_*)^2^ × [*P*(1-*P*)]/*δ*^2^ as 85 cases (12). A total of 172 cases were included in this study, which met the statistical power requirements. This study was approved by the Ethics Committee of the hospital (Approval No.: [2026] Lunshenyanlin No. (02)).

Definition and Grouping of Inflammatory and Nutritional Parameters

All enrolled patients provided fasting peripheral venous blood samples within one week prior to initiating immunotherapy. Complete blood count and serum albumin levels were measured to obtain neutrophil, lymphocyte, platelet, and monocyte counts. Calculations were performed as follows: NLR = neutrophil count/lymphocyte count; PLR = platelet count/lymphocyte count; LMR = lymphocyte count/monocyte count. Nutritional Risk Index (NRI) was calculated using a modified formula:: NRI = 1.519 × serum albumin (g/L) + 0.417 × (actual body weight/ideal body weight  ×  100), with ideal body weight (kg) determined as height (cm) - 105.

With patient mortality as the outcome variable, ROC curves were plotted and the Youden index was computed to identify the optimal cut-off values for NLR, PLR, LMR and NRI. Study participants were subsequently stratified into low- and high-value subgroups based on these defined cut-off values.

### Treatment regimens

All patients received immune checkpoint inhibitor treatment. PD-1 monoclonal antibodies were intravenously infused every 3 weeks as a cycle; including pembrolizumab 200 mg, sintilimab 200 mg, or tislelizumab 200 mg. For patients receiving combination therapy: ① Combined with anti-angiogenic therapy: bevacizumab 7.5 mg/kg or 10 mg/kg intravenously infused once every 3 weeks, synchronized with the immunotherapy cycle; ② Combined with chemotherapy: capecitabine 1000 mg/m^2^ orally twice a day for 14 consecutive days, with 7 days of rest as a 3-week cycle, or mFOLFOX6 regimen (oxaliplatin 85 mg/m^2^ intravenously on day 1, leucovorin 400 mg/m^2^ intravenously on day 1, fluorouracil 400 mg/m^2^ bolus on day 1, then 1200 mg/m^2^/day continuous infusion for 46 hours) every 2 weeks as a cycle. During treatment, the dose was adjusted or treatment was suspended according to the severity of adverse reactions graded by the National Cancer Institute Common Terminology Criteria for Adverse Events (NCI-CTCAE) version 5.0, and symptomatic treatment was given for adverse reactions.

### Outcome measures

#### Data collection

General data (age, gender, etc.), clinicopathological data (TNM staging, treatment regimens, etc.), laboratory test indicators (neutrophil count, lymphocyte count, monocyte count, platelet count, serum albumin), and efficacy evaluation results of patients were collected.

#### Efficacy and prognosis evaluation

(1) Efficacy was evaluated using the RECIST v1.1 standard (11) at T1 time point, which was divided into complete response (CR), partial response (PR), stable disease (SD), and progressive disease (PD). ORR = (number of CR + PR cases)/total number of cases  ×  100%, disease control rate (DCR) = (number of CR + PR + SD cases)/total number of cases  ×  100%.
(2) PFS and OS were defined as the core endpoints for evaluating long-term therapeutic efficacy. PFS was calculated as the time from the date of treatment initiation to the first occurrence of disease progression or death from any cause. OS was defined as the time interval from the date of treatment initiation to death from any cause. During follow-up, the occurrence of disease progression or death was recorded as the time point of the endpoint event. For patients without the corresponding endpoint event, the date of 36 months after the first treatment was set as the censoring endpoint. After completing the prescribed immunotherapy regimen, patients underwent contrast-enhanced abdominopelvic computed tomography (CT)/magnetic resonance imaging (MRI) every 3 months. The survival status, tumor progression, and subsequent treatment regimens were concurrently documented until the follow-up termination criteria were met. Disease progression was independently confirmed by two attending physicians from the medical oncology and radiology departments in accordance with the RECIST v1.1 criteria (11).

### Statistical analysis

All statistical analyses were conducted using SPSS 26.0 software. Continuous variables were presented as mean ± standard deviation, with intergroup comparisons performed via the t-test or analysis of variance (ANOVA); categorical variables were expressed as *n* (%), and intergroup differences were examined using the chi-square test or Fisher's exact test. Standard ROC curve analysis was applied to determine the optimal cut-off values for NLR, PLR, LMR and NRI, and time-dependent ROC curve analysis was performed to evaluate the predictive performance of indicators for 12-month and 24-month PFS and OS, with AUC and 95%CI calculated. Survival curves were generated with the Kaplan–Meier method, and the Log-rank test was used to compare differences in PFS and OS among patient subgroups stratified by the above parameters. Univariate Cox proportional hazards regression analysis was performed to identify risk factors associated with patients’ PFS and OS; variables with a *P* value < 0.05 in the univariate analysis were subsequently incorporated into the multivariate Cox proportional hazards regression model using the forward stepwise (Likelihood Ratio) method to screen for independent prognostic factors. Variance Inflation Factor (VIF) test was performed to assess multicollinearity among variables included in the Cox model, and variables with VIF >5 were excluded to ensure model stability. Bootstrap method with 1000 repeated samplings was used for internal validation of the multivariate model. A two-sided *P* < 0.05 was considered to indicate a statistically significant difference.

## Results

### Baseline characteristics of patients

A total of 172 patients with advanced rectal cancer were included in this study. The cohort comprised 102 males (59.30%) and 70 females (40.70%), with ages ranging from 42 to 88 years (mean age: 65.2 ± 10.3 years). Regarding TNM staging, 98 patients (56.98%) were classified as stage Ⅲ and 74 (43.02%) as stage Ⅳ, among whom 92 patients (53.49%) presented with distant metastasis. Among the 98 stage Ⅲ patients, 72 cases were inoperable due to locally advanced tumors invading adjacent organs/pelvic wall, and 26 cases were inoperable due to severe comorbidities that cannot tolerate radical surgery. A total of 92 patients (53.49%) presented with distant metastasis at baseline, including liver metastasis in 48 cases (52.17%), lung metastasis in 22 cases (23.91%), peritoneal metastasis in 12 cases (13.04%), bone metastasis in 6 cases (6.52%), and other distant organ metastasis (including brain, adrenal gland, etc.) in 4 cases (4.35%). Among them, 18 patients had two or more distant metastatic sites. In terms of tumor differentiation, 119 cases (69.19%) were poorly differentiated, while 53 cases (30.81%) were moderately to well differentiated. Treatment regimens consisted of immunotherapy alone in 71 patients (41.28%) and combined immunotherapy in 101 patients (58.72%). Efficacy evaluation revealed the following outcomes: CR in 10 patients (5.81%), PR in 41 patients (23.84%), SD in 68 patients (39.53%), and PD in 53 patients (30.81%). Consequently, the ORR was 29.65%, and the DCR reached 69.18%. At the end of follow-up, a total of 128 all-cause deaths were recorded in the cohort. The median OS of the entire cohort was 22.5 months (95%CI: 19.2-25.8 months), with 12-month and 24-month OS rates of 76.2% and 48.3%, respectively. The median PFS of the entire cohort was 18.0 months (95%CI: 14.6-21.4 months), with 12-month and 24-month PFS rates of 62.8% and 37.2%, respectively.

The distribution of inflammatory and nutritional parameters before treatment was as follows: neutrophil count (4.86 ± 1.52) × 10^9^/L, lymphocyte count (1.68 ± 0.57) × 10^9^/L, platelet count (265.42 ± 85.36) × 10^9^/L, monocyte count (0.42 ± 0.15) × 10^9^/L, serum albumin (38.65 ± 5.21) g/L, NLR (3.12 ± 1.25), PLR (156.38 ± 42.65), LMR (4.05 ± 1.12), NRI (50.12 ± 6.35) (Table 1).

**Table 1:** Baseline characteristics of patients [n(%)/ $\bar{\chi }\pm s$ ].

Indicator	n(%)/$\bar{\chi }\pm s$	Indicator	n(%)/$\bar{\chi }\pm s$
Gender		Tumor Differentiation Grade	
Male	102（59.30）	Poorly-undifferentiated	119（69.19）
Female	70（40.70）	Well-moderately differentiated	53（30.81）
Age (years)		TNM Stage	
≥60	104（60.47）	Ⅲ	98（56.98）
<60	68（39.53）	Ⅳ	74（43.02）
Neutrophil count (×10^9^/L)	4.86 ± 1.52	Treatment Regimen	
Lymphocyte count (×10^9^/L)	1.68 ± 0.57	Monotherapy	71（41.28）
Platelet count (×10^9^/L)	265.42 ± 85.36	Combination Therapy	101（58.72）
Monocyte count (×10^9^/L)	0.42 ± 0.15	Clinical Efficacy	
Serum albumin (g/L)	38.65 ± 5.21	CR	10（5.81）
NLR	3.12 ± 1.25	PR	41（23.84）
PLR	156.38 ± 42.65	SD	68（39.53）
LMR	4.05 ± 1.12	PD	53（30.81）
NRI	50.12 ± 6.35	ORR	51（29.65）
Distant Metastasis		DCR	119（69.18）
Yes	92（53.49）		
No	80（46.51）		

NLR, Neutrophil-to-Lymphocyte Ratio; PLR, Platelet-to-Lymphocyte Ratio; LMR, Lymphocyte-to-Monocyte Ratio; NRI, Nutritional Risk Index; CR, complete response; PR, partial response; SD, stable disease; PD, progressive disease; ORR, objective response rate; DCR, disease control rate (DCR).

### Optimal Cut-off values of parameters determined by ROC curves

**Figure 1:**
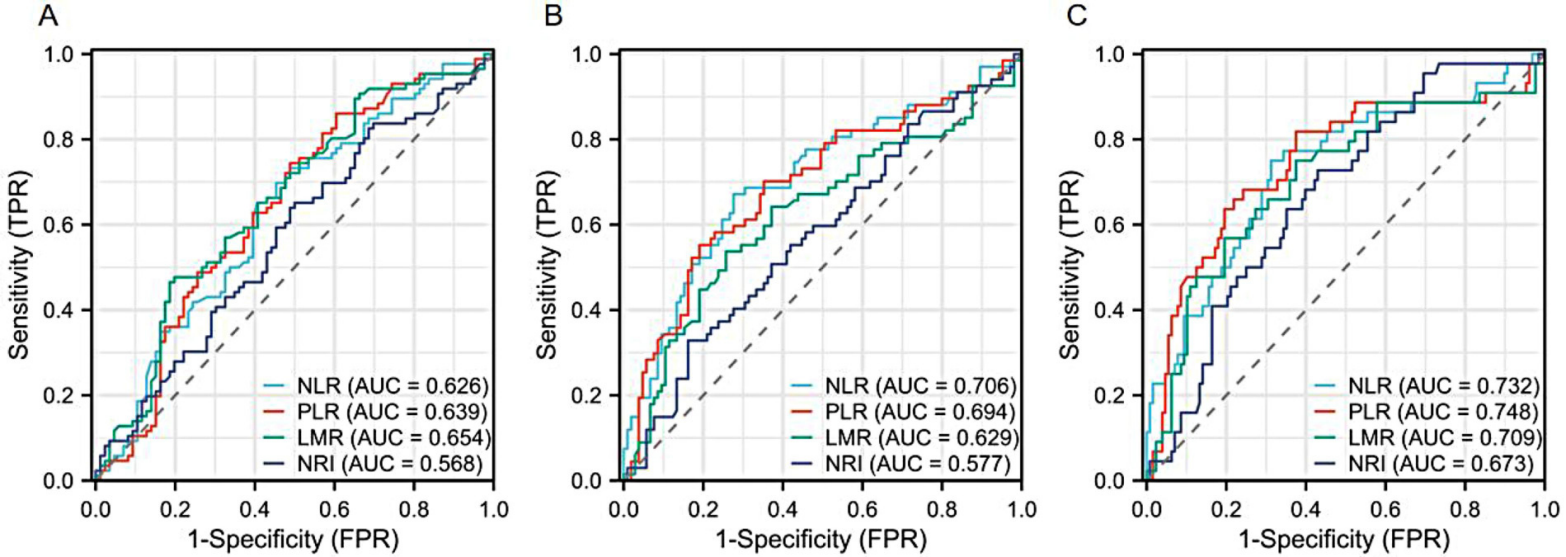
Time-dependent ROC curves for survival prediction in rectal cancer patients. **(A)** 12 months; **(B)** 24 months; **(C)** 36 months.

Time-dependent ROC curves were used to evaluate the dynamic predictive performance of the four indicators (NLR, PLR, LMR, and NRI) for patients’ OS at 12, 24, and 36 months, as shown in Table 2 and Figure 1. At 12 months, the AUC of NLR, PLR, LMR, and NRI for predicting OS was 0.626, 0.639, 0.654, and 0.568, respectively; at 24 months, the AUC of the four indicators for OS prediction increased to 0.706, 0.694, 0.629, and 0.577, respectively; at 36 months, the AUC of the four indicators was 0.732, 0.748, 0.709, and 0.673, respectively. Time-dependent ROC curve analysis showed that all four indicators (NLR, PLR, LMR, and NRI) exhibited moderate predictive performance at each time point.

**Table 2:** Time-dependent ROC curve analysis of NLR, PLR, LMR and NRI for predicting overall survival in advanced rectal cancer.

Indicator	Time Point	AUC	Sensitivity (%)	Specificity (%)	95% CI	cut-off	Youden Index
NLR	12 months	0.626	51.16	62.21	0.542–0.710	2.36	0.244
	24 months	0.706	67.16	72.38	0.624–0.789	2.42	0.395
	36 months	0.732	75.00	68.75	0.642–0.823	2.50	0.437
PLR	12 months	0.639	51.16	62.79	0.555–0.722	112.54	0.256
	24 months	0.694	55.22	80.95	0.610–0.778	122.95	0.362
	36 months	0.748	81.82	62.50	0.654–0.841	142.23	0.443
LMR	12 months	0.654	47.67	80.23	0.572–0.737	3.36	0.221
	24 months	0.629	53.73	74.29	0.539–0.718	3.45	0.280
	36 months	0.709	75.00	62.50	0.613–0.805	3.92	0.375
NRI	12 months	0.568	32.84	83.81	0.483–0.654	42.16	0.166
	24 months	0.577	63.95	51.16	0.489–0.665	48.57	0.279
	36 months	0.673	72.73	57.03	0.586–0.761	50.45	0.297

The optimal cut-off values were determined by the maximum Youden index at 36 months, as follows: NLR = 2.50 (Youden index = 0.437, 75.00% sensitivity, 68.75% specificity), PLR = 142.23 (Youden index = 0.443, 81.82% sensitivity, 62.50% specificity), LMR = 3.92 (Youden index = 0.375, 75.00% sensitivity, 62.50% specificity) and NRI = 50.45 (Youden index = 0.297, 72.73% sensitivity, 57.03% specificity). Patients were then stratified into high/low subgroups by these cut-offs: high NLR (≥2.50, *n* = 68)/low NLR (<2.50, *n* = 104); high PLR (≥142.23, *n* = 71)/low PLR (<142.23, *n* = 101); low LMR (≤3.92, *n* = 95)/high LMR (>3.92, *n* = 77); low NRI (≤50.45, *n* = 89)/high NRI (>50.45, *n* = 83) (Figure 1A).

### Correlations between inflammatory, nutritional parameters and clinicopathological characteristics

Table 3 revealed that high NLR, high PLR, low LMR and low NRI were significantly associated with tumor differentiation, TNM stage and distant metastasis (*P* < 0.05), but not with gender, age or treatment regimen (*P* > 0.05). Specifically, the proportions of high NLR, high PLR, low LMR and low NRI were significantly higher in patients with poorly differentiated tumors than in those with well-moderately differentiated ones; the same abnormal indicator proportions were markedly higher in stage Ⅳ than stage Ⅲ patients, and in patients with distant metastasis than those without.

**Table 3:** Correlations between inflammatory, nutritional parameters and clinicopathological characteristics of patients with advanced rectal cancer [*n*(%)].

Variable		NLR			PLR			LMR			NRI		
	n	High (*n* = 68)	Low (*n* = 104)	*χ* ^2^ */P*	High (*n* = 71)	Low (*n* = 101)	*χ* ^2^ */P*	Low (*n* = 95)	High (*n* = 77)	*χ* ^2^ */P*	Low (*n* = 89)	High (*n* = 83)	*χ* ^2^ */P*
Gender				0.528/0.468			0.002/0.964			1.015/0.314			0.128/0.720
Male	102	38 (55.88)	64 (61.54)		42 (59.15)	60 (59.41)		53 (55.79)	49 (63.64)		48 (62.34)	54 (65.06)	
Female	70	30 (44.12)	40 (38.46)		29 (40.85)	41 (40.59)		42 (44.21)	28 (36.36)		41 (46.07)	29 (34.94)	
Age (years)				0.785/0.375			0.362/0.547			0.018/0.894			2.914/0.088
≤60	68	24 (35.29)	44 (42.31)		26 (36.62)	42 (41.58)		38 (40.00)	30 (38.96)		41 (46.07)	27 (32.53)	
>60	104	44 (64.71)	60 (57.69)		45 (63.38)	59 (58.42)		57 (60.00)	47 (61.04)		48 (53.93)	56 (67.47)	
TNM stage				32.651/<0.001			29.874/<0.001			38.526/<0.001			35.128/<0.001
Ⅲ	98	20（29.41）	78（75.00）		22 (30.99)	76 (75.25)		33（34.74）	65（84.42）		31（34.83）	67（80.72）	
Ⅳ	74	48（70.59）	26（25.00）		49 (69.01)	25 (24.75)		62（65.26）	12（15.58）		58（65.17）	16（19.28）	
Distant metastasis				12.365/<0.001			10.582/0.001			11.257/<0.001			9.863/0.002
Yes	92	48 (70.59)	44 (42.31)		49 (69.01)	43 (42.57)		62 (65.26)	30 (38.96)		58 (65.17)	34 (41.00)	
No	80	20 (29.41)	60 (57.69)		22 (30.99)	58 (57.43)		33 (34.74)	47 (61.04)		31 (34.83)	49 (59.00)	
Tumor differentiation				8.562/0.003			13.254/<0.001			16.325/<0.001			11.658/<0.001
Poorly-undifferentiated	119	56 (82.35)	63 (60.58)		61 (85.92)	58 (57.43)		79 (83.16)	40 (51.95)		72 (80.90)	47 (56.63)	
Well-moderately differentiated	53	12 (17.65)	41 (39.42)		10 (14.08)	43 (42.57)		16 (16.84)	37 (48.05)		17 (19.10)	36 (43.37)	
Treatment regimen				1.526/0.217			2.015/0.156			0.752/0.437			2.365/0.124
Monotherapy	71	32 (47.06)	39 (37.50)		34 (47.89)	37 (36.63)		42 (44.21)	29 (37.66)		42 (47.19)	29 (34.94)	
Combined therapy	101	36 (52.94)	65(62.50)		37(52.11)	64(63.37)		53(55.79)	48(62.34)		47(52.81)	54(65.06)	

### Correlations between inflammatory, nutritional parameters and immunotherapy efficacy

Immunotherapy efficacy differed significantly across the four parameter subgroups (all *P* < 0.05, Table 4). The low NLR group had notably higher ORR (39.42%) and DCR (81.73%) than the high NLR group (14.71%, 50.00%); the low PLR group had higher ORR (38.61%) and DCR (79.21%) than the high PLR group (16.90%, 54.93%); the high LMR group had higher ORR (48.05%) and DCR (81.82%) than the low LMR group (14.74%, 58.95%); the high NRI group had higher ORR (44.58%) and DCR (79.52%) than the low NRI group (15.73%, 59.55%).

**Table 4:** Correlations between inflammatory, nutritional parameters and immunotherapy efficacy in patients with advanced rectal cancer [*n*(%)].

Group	n	CR	PR	SD	PD	ORR(%)	*P* < 0.001	DCR(%)	*P* < 0.001
NLR									
High	68	5 (7.35)	5 (7.35)	24 (35.29)	34 (50.00)	14.71		50.00	
Low	104	5 (4.81)	36 (34.62)	44 (42.31)	19 (18.27)	39.42		81.73	
PLR							<0.001		<0.001
High	71	2 (2.82)	10 (14.08)	27 (38.03)	32 (45.07)	16.90		54.93	
Low	101	8 (7.92)	31 (30.69)	41 (40.59)	21 (20.79)	38.61		79.21	
LMR							<0.001		<0.001
High	77	7 (9.09)	30 (38.96)	26 (33.77)	14 (18.18)	48.05		81.82	
Low	95	3 (3.16)	11 (11.58)	42 (44.21)	39 (41.05)	14.74		58.95	
NRI							<0.001		<0.001
High	83	7 (8.43)	30 (36.14)	29 (34.94)	17 (20.48)	44.58		79.52	
Low	89	3 (3.37)	11 (12.36)	39 (43.82)	36(40.45)	15.73		59.55	

### Relationships between inflammatory, nutritional parameters and patient survival prognosis

**Figure 2:**
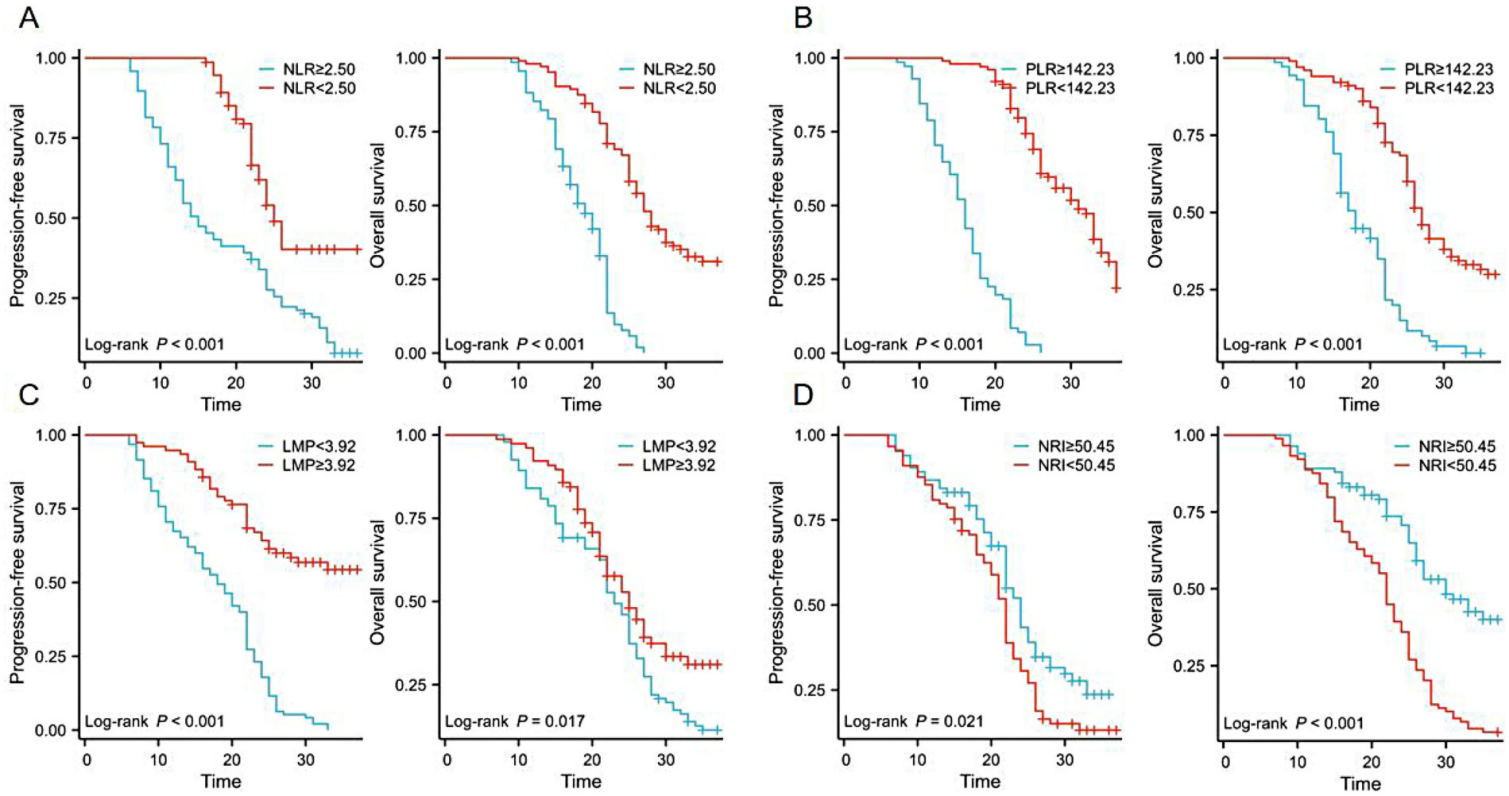
PFS and OS survival curves of patients in different inflammatory and nutritional parameter groups [note: **A∼D** are PFS (left) and OS (right) curves of NLR, PLR, LMR, and NRI groups, respectively].

Kaplan–Meier survival analysis revealed that the low NLR group had markedly longer median PFS (25 months) and OS (27months) than the high NLR group (15 and 19 months, respectively, *P* all < 0.001); the low PLR group also presented significantly longer median PFS (31 months) and OS (27 months) compared with the high PLR group (16 and 18 months, respectively, *P* all < 0.001). For LMR, the high subgroup had slightly longer median PFS (26 months) and OS (25 months) than the low subgroup (18 and 23 months, *P* < 0.001 and *P* = 0.017, respectively); for NRI, the low subgroup had marginally longer median PFS (22 months) and OS (22 months) than the high subgroup (24 and 30 months, *P* *=* 0.021 and <0.001) (Figure 2).

### Univariate and multivariate cox regression analyses of factors affecting PFS and OS

**Figure 3:**
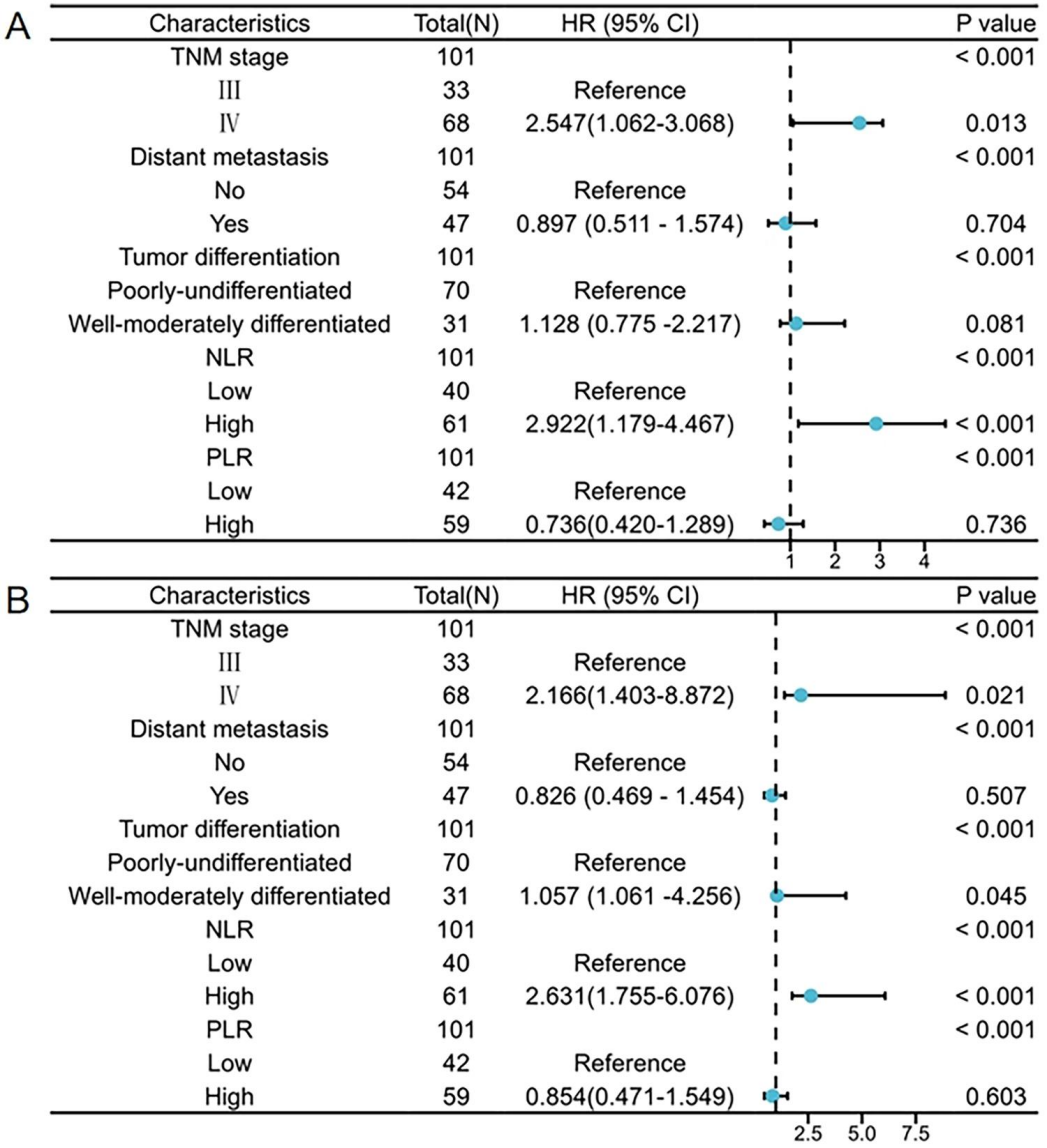
Forest plots of multivariate Cox regression analysis in patients with advanced rectal cancer [**(A)**: PFS; **(B)**: OS].

VI*F* test showed that the VIF values of all variables included in the Cox regression model were <2, indicating no significant multicollinearity and stable model structure. Bootstrap internal validation with 1000 repeated samplings showed that the HR values of the multivariate model were consistent with the original results, with C-index of 0.712 for PFS and 0.705 for OS, confirming the robustness of the model. Cox regression revealed that TNM stage Ⅳ and high NLR (≥2.50) were independent risk factors for shortened PFS (Table 5, Figure 3A). For OS, TNM stage Ⅳ, poorly differentiated tumors and high NLR (≥2.50) were confirmed as independent risk factors for reduced survival time (Table 6, Figure 3B).

**Table 5:** Univariate and multivariate Cox regression analyses of factors affecting PFS in patients with advanced rectal cancer.

Variable	Univariate Cox regression				Multivariate Cox regression		
	HR	95% CI	*P*	VIF	HR	95% CI	*P*
Gender	1.215	0.826–1.789	0.318	1.136	-	-	-
Age	1.032	0.698–1.524	0.865	1.077	-	-	-
TNM stage	2.864	1.925–4.258	<0.001	1.213	2.014	1.326–3.057	0.001
Distant metastasis	2.105	1.432–3.098	<0.001	1.306	1.127	0.715–1.776	0.602
Tumor differentiation	2.357	1.586–3.504	<0.001	1.414	1.203	0.784–1.844	0.396
Treatment regimen	1.153	0.786–1.692	0.465	1.049	-	-	-
NLR	3.865	2.624–5.697	<0.001	1.240	2.689	1.772–4.081	<0.001
PLR	2.415	1.632–3.568	<0.001	1.213	1.084	0.695–1.691	0.723
LMR	0.942	0.635–1.397	0.756	1.174	-	-	-
NRI	0.815	0.548–1.212	0.315	1.220	-	-	-

Endpoint events for PFS: 163 cases. Variable assignment: Sex (male, 1; female, 0); Age (>60 years, 1, ≤60 years, 0); TNM stage (stage Ⅳ, 1; stage Ⅲ, 0); Distant metastasis (yes, 1; no, 0); Tumor differentiation (poor-undifferentiated, 1; well-moderately differentiated, 0); Treatment regimen (combination therapy, 1; immunotherapy monotherapy, 0); NLR (≥2.50, 1; <2.50, 0); PLR (≥142.23, 1; <142.23, 0); LMR (≤3.92,  1; >3.92, 0); NRI (≤50.45, 1; >50.45, 0).

**Table 6:** Univariate and multivariate Cox regression analyses of factors affecting OS in patients with advanced rectal cancer.

Variable	Univariate Cox regression				Multivariate Cox regression		
	HR	95% CI	*P*	VIF	HR	95% CI	*P*
Gender	1.196	0.812–1.763	0.342	1.136	-	-	-
Age	1.018	0.685–1.510	0.926	1.077	-	-	-
TNM stage	2.735	1.842–4.056	<0.001	1.171	1.923	1.231–3.004	0.004
Distant metastasis	2.057	1.398–3.024	<0.001	1.307	1.075	0.678–1.704	0.757
Tumor differentiation	2.468	1.657–3.679	<0.001	1.372	1.786	1.168–2.730	0.007
Treatment regimen	1.132	0.772–1.658	0.513	1.068	-	-	-
NLR	3.652	2.486–5.368	<0.001	1.631	2.519	1.648–3.852	<0.001
PLR	2.368	1.597–3.512	<0.001	1.216	1.062	0.681–1.657	0.789
LMR	0.927	0.625–1.375	0.702	1.165	-	-	-
NRI	0.796	0.535–1.185	0.263	1.215	-	-	-

Total endpoint events for OS: 128 cases. Variable assignment: Sex (male, 1; female, 0); Age (>60 years, 1; ≤60 years, 0); TNM stage (stage Ⅳ, 1, stage Ⅲ, 0); Distant metastasis (yes, 1; no, 0); Tumor differentiation (poor-undifferentiated, 1; well-moderately differentiated, 0); Treatment regimen (combination therapy, 1; immunotherapy monotherapy, 0); NLR (≥2.50, 1; <2.50, 0); PLR (≥142.23, 1; <142.23, 0); LMR (≤3.92, 1, >3.92, 0); NRI (≤50.45, 1; >50.45, 0).

## Discussion

Rectal cancer is a frequent malignant tumor of the gastrointestinal tract that has a high incidence and fatality rate worldwide (13). Patients with severe illness have few therapeutic options and a dismal outlook. The ORR of only about 30% and the high treatment cost underscore the clinical urgency of finding useful predictive biomarkers, despite the fact that immunotherapy based on PD-1/PD-L1 inhibitors has completely changed the therapeutic paradigm of advanced rectal cancer. Nutritional status and systemic inflammatory response are key factors affecting immune function and treatment response in tumor patients. In order to provide empirical support for the creation of customized treatment plans, this study concentrated on four readily available clinical indicators (NRI, NLR, PLR, and LMR) and methodically investigated their predictive value.

The AUC values of the four inflammatory and nutritional indicators for OS prediction in this study ranged from 0.673 to 0.748, showing a moderate level of predictive efficacy. As non-tumor-specific markers, peripheral blood inflammatory and nutritional indicators are affected by systemic physiological and pathological conditions such as nutritional status, underlying diseases, and non-tumor inflammatory response, so the discriminatory ability of a single indicator is naturally limited, which is consistent with the results of most similar studies on tumor immunotherapy (14). Tumor-related inflammation plays a central role in immunotherapy resistance by regulating the immunosuppressive microenvironment, promoting tumor angiogenesis, invasion, and metastasis (9). As a key indicator reflecting the balance between neutrophils and lymphocytes, an elevated NLR implies enhanced neutrophil-mediated inflammatory response and impaired lymphocyte anti-tumor immune function (15). ROC curve analysis determined an optimal NLR cutoff value of 2.50 in this study. The optimal cut-off value of NLR was determined as 2.50 via ROC curve analysis in our study. This value is consistent with the NLR cut-off of 2.8 reported in previous similar studies, yet slightly lower than the widely accepted range of 3.0–5.0 for other malignancies. This discrepancy may be attributed to three potential reasons: First, most studies with a higher NLR cut-off focused on early-stage resectable tumors, while our study enrolled patients with advanced rectal cancer, who had significantly different baseline inflammatory and immune status from early-stage patients (16); Second, our study focused on patients treated with immunotherapy, whereas most previous studies targeted patients undergoing chemotherapy or surgery, and the correlation between NLR and treatment response may vary by treatment modality (17); Third, all enrolled patients were Chinese, and the baseline distribution of peripheral blood cell counts may differ from that of Western populations, resulting in the variance of the optimal cut-off value. Multivariate Cox regression further confirmed that high NLR (≥2.50) was the strongest independent risk factor for shortened PFS and OS, with predictive efficacy significantly superior to other indicators. This result is consistent with findings by Xu et al. (14), suggesting that elevated NLR may disrupt the stability of the tumor microenvironment through substances such as matrix metalloproteinases and reactive oxygen species released by neutrophils, inhibiting T-cell infiltration and function, thereby weakening the immunotherapeutic response.

Patients in the high PLR group exhibited significantly inferior ORR, DCR and survival outcomes compared with those in the low PLR group. By producing cytokines like platelet-derived growth factor and transforming growth factor-*β*, platelets can aid in tumor cell proliferation, angiogenesis, and immune escape in addition to their coagulatory roles (18). Nevertheless, PLR was not identified as an independent risk factor in multivariate analysis, implying that its predictive value may depend on synergistic effects with NLR or be confounded by factors like tumor stage and differentiation degree. This finding is consistent with previous studies that regard PLR as a secondary predictive indicator (19, 20).

LMR reflects the balance between lymphocytes and monocytes. Lymphocytes are core effector cells of anti-tumor immunity, while monocytes can differentiate into immunosuppressive macrophages involved in tumor immune escape (21). The high LMR group (>3.92) had markedly higher ORR (48.89% vs 14.29%) and DCR (82.22% vs 58.93%) than the low LMR group, yet no statistically significant differences in PFS and OS were detected between the two groups in survival analysis. Previous studies have highlighted the prognostic role of LMR in colorectal cancer. A study published in the journal Medicine, titled ‘Preoperative Prognostic Value of Lymphocyte-to-Monocyte Ratio in Patients with Recurrent Colorectal Cancer’, demonstrated that low preoperative LMR was independently associated with poorer survival in recurrent colorectal cancer patients (22). These findings underscore the biological importance of the lymphocyte-monocyte balance in tumor progression and immune regulation. Although LMR was not identified as an independent prognostic factor in our multivariate analysis, its association with ORR and DCR suggests that baseline immune-inflammatory status may still influence immunotherapy responsiveness. Differences in disease stage, treatment modality (surgery vs. immunotherapy), and patient selection may partly explain discrepancies between studies. Subsequent verification through larger-sample clinical investigations is thus required for this result.

Nutritional status is the foundation for maintaining immune function and treatment tolerance in tumor patients. As a core nutritional indicator, serum albumin directly reflects the body's protein reserves and inflammatory stress levels, while weight changes reflect the balance between long-term nutritional intake and consumption (23). This result verifies the important impact of nutritional status on immunotherapeutic response: a good nutritional status can maintain lymphocyte proliferation activity and cytokine secretion function, enhancing the anti-tumor immune response mediated by PD-1/PD-L1 inhibitors; in contrast, malnutrition downregulates immune cell function and induces an inflammatory cytokine storm, exacerbating immunotherapy resistance (4, 5). Although univariate analysis showed a correlation between NRI and survival outcomes, it did not emerge as an independent risk factor in multivariate analysis. This may be because nutritional status is influenced by multiple factors such as tumor progression and treatment intervention, and its predictive value is weakened when combined with clinical indicators such as TNM stage and tumor differentiation degree.

Multivariate analysis in this study confirmed that TNM stage Ⅳ was an independent risk factor for shortened PFS (HR = 2.014, *P* = 0.001) and OS (HR = 1.923, *P* = 0.004), consistent with the natural course of advanced rectal cancer: stage Ⅳ patients are often accompanied by distant metastasis, with higher tumor burden and a more complex immune microenvironment, making it difficult for immunotherapy to effectively eliminate systemic lesions (24). In addition, poorly-undifferentiated tumor status was an independent risk factor for shortened OS (HR = 1.786, 95%CI: 1.168-2.730, *P* = 0.007). Clinically, poorly differentiated tumors are characterized by high proliferative activity, strong invasiveness, and higher risk of distant metastasis, which may lead to poorer long-term prognosis even after immunotherapy. Poorly-differentiated tumor cells exhibit high proliferative activity and invasiveness, and are often associated with higher levels of inflammatory factor secretion, further exacerbating the formation of an immunosuppressive microenvironment (25). No significant prognostic difference was observed between treatment regimens, which may be related to the higher baseline tumor burden in patients receiving combined therapy. Further verification of the beneficiary population for combined therapy through propensity score matching is required.

The strengths of this study include: ① The sample size meets statistical power requirements with complete follow-up, ensuring high reliability of results; ② Focus on routine clinical detection indicators without additional testing costs, resulting in strong operability; ③ Identification of optimal cut-off values for each indicator via ROC curve analysis, combined with univariate and multivariate analyses to systematically screen independent predictive factors, demonstrating clear clinical transformation value.

## Limitations

While the present study has its merits, it is not without limitations. This study was a single-center retrospective study, with enrolled patients receiving different types of PD-1 inhibitors; some patients also received chemotherapy or anti-angiogenic therapy in combination. The heterogeneity of treatment regimens may have a certain impact on the study results, and subsequent prospective studies need to standardize treatment regimens to reduce confounding bias. The sample size of 172 patients, while meeting the statistical power requirements, is still relatively small for subgroup analysis, and there may be type II error for the negative results of LMR and NRI, which need to be verified in a larger cohort. This study only focused on the baseline levels of inflammatory and nutritional indicators before treatment, lacking dynamic monitoring of indicator changes during treatment, while dynamic fluctuations may better reflect treatment response and prognosis. The enrolled patients received different types of PD-1 inhibitors and combination therapy regimens, and the treatment heterogeneity may have a certain impact on the results. The predictive performance of single inflammatory indicators is moderate, and the clinical application value as an independent biomarker is limited, which needs to be combined with other clinical and molecular markers to improve the predictive efficacy. This study did not collect the MSI status of enrolled patients, which is a key biomarker for rectal cancer immunotherapy, and the interaction between MSI status and inflammatory indicators needs to be further explored. This study did not complete the construction and validation of a multi-indicator combined prediction model. Subsequent studies can build a visualized prediction model by integrating independent prognostic factors based on the results of this study, and complete internal and external validation.

Future research could conduct multi-center prospective cohort studies to validate the cut-off values and combined prediction models identified in this study; guide real-time adjustment of treatment regimens through dynamic monitoring of indicator changes during treatment; and explore the molecular mechanisms by which indicators such as NLR affect immunotherapeutic efficacy using techniques such as single-cell sequencing and flow cytometry, providing an empirical framework for the creation of targeted intervention tactics.

## Conclusion

In summary, pretreatment NRI, NLR, PLR, and LMR are closely correlated with the efficacy and prognosis of PD-1/PD-L1 inhibitor immunotherapy in patients with advanced rectal cancer. Among them, NLR≥2.50 can be used as an independent risk factor for poor immunotherapy efficacy and unfavorable prognosis, with moderate predictive performance. These indicators are simple, economical, and easy to detect, which can be used as auxiliary tools for clinical evaluation of immunotherapy benefit and prognosis, providing a reliable reference for clinical screening of populations benefiting from immunotherapy and formulating individualized treatment plans. Their clinical application value still needs to be further verified in multi-center, large-sample prospective cohorts.

## Data Availability

The original contributions presented in the study are included in the article/supplementary material, further inquiries can be directed to the corresponding author.
